# Comparative Effectiveness of Radiofrequency Ablation vs. Surgical Resection for Patients With Solitary Hepatocellular Carcinoma Smaller Than 5 cm

**DOI:** 10.3389/fonc.2020.00399

**Published:** 2020-03-31

**Authors:** Lei Zheng, Chi-Hao Zhang, Jia-Yun Lin, Chen-Lu Song, Xiao-Liang Qi, Meng Luo

**Affiliations:** ^1^Department of General Surgery, School of Medicine, Shanghai Ninth People's Hospital, Shanghai Jiao Tong University, Shanghai, China; ^2^Department of Burns and Plastic Surgery, School of Medicine, Shanghai Ninth People's Hospital, Shanghai Jiao Tong University, Shanghai, China

**Keywords:** hepatocellular carcinoma, radiofrequency ablation, surgical resection, overall survival, disease-free survival

## Abstract

**Background:** This study aims to compare survival outcome after receiving radiofrequency ablation (RFA) and surgical resection (SR) for solitary hepatocellular carcinoma (HCC) with size large as 5 cm.

**Methods:** The SEER database was queried for patients with HCC tumors who were treated with RFA or SR between 2004 and 2015. Univariate and multivariate Cox analysis was used to assess the influence of potential variables on the patients' outcome. Additionally, propensity score matching (PSM) and multiple imputations (MI) were used as sensitivity analyses.

**Results:** Of 1,985 cases, 934 patients received RFA treatment, while the rest underwent surgical resection. The patients in the RFA group had poorer overall survival (OS) and cancer-specific survival (CSS) than those in the SR group regardless of the tumor size before matching and MI. By using PSM analysis at a 1:1 ratio, 1,302 cases were paired and we have found that SR had a positive impact on OS and CSS of patients with tumors measuring from 3.1 to 5 cm. However, when the tumor size was <3 cm, patients undergoing SR had similar survival benefit with those after RFA. The above results were confirmed after performing PSM analysis at a 1:2 and 1:3 ratio.

**Conclusion:** By applying several effective sensitivity analyses, we demonstrated that OS and CSS were similar between the patients with tumors smaller than 3 cm receiving RFA and SR. But SR may be a superior treatment option with better long-term outcome than RFA in patients with tumor measuring 3.1–5 cm.

## Introduction

Hepatocellular carcinoma (HCC) is the fifth most frequent cancer and the third most common cause leading to cancer-related mortality worldwide (1, 2). It is estimated that ~500,000 deaths from HCC occur per year. At diagnosis, no more than 20% of patients are ultimately eligible for curative treatments, such as liver transplantation, surgical removal, and radiofrequency ablation (RFA), mainly due to the presence of metastatic sites or heavy tumor burden (3). Liver transplantation is regarded as the best choice of therapy if possible, as it also treats the remaining liver that is most often cirrhotic. The Milan criteria (4–6), the standard for liver transplant eligibility, are defined as a solitary nodule ≤ 5 cm, or up to 3 nodules ≤ 3 cm, with no evidence of vascular invasion, and enough liver functional reserve. But owing to the shortage of available liver donors, this technique is limited in clinical practice and only a few patients have the chance to accept this kind of treatment. For those with one tumor ≤ 5 cm, who are suitable for transplants but with a low likelihood of receiving an organ, surgical resection (SR) and radiofrequency ablation (RFA) have been suggested as a first-line treatment option.

Currently, there are many studies that have investigated the efficacy of these two therapies (2, 4, 7, 8). But it is still controversial whether RFA or SR results in more favorable treatment outcomes for patients with small lesions. To the best of our knowledge, three randomized trials have been conducted on this issue and the results were discordant. Two of them have reported that SR was similar to RFA in terms of overall survival (OS) (9, 10), while the third one demonstrated that SR offered better OS and disease-free survival (DFS) (6). These results could be explained by the different tumor sizes chosen for RFA and SR treatment.

Although RFA is proposed as preferred therapy in treating small HCC, it is still unclear the maximum HCC tumor size at which RFA continues to be safe and effective. Some proposed that tumor size measuring up to 3 cm was an indication for RFA treatment for HCC (11). However, a multi-center study conducted by Italian scientists found that for tumors smaller than 2 cm, there is no significant survival difference between RFA and SR (5). Furthermore, another study found that even for tumors up to 5 cm, RFA is still effective and can be applied as the first-line treatment (12). Because the therapeutic efficiency of RFA and SR are different in the setting of different tumor size, there is clinical confusion when considering which approach is better for patients. Therefore, to clarify this issue, we stratified patients based on the above tumor size cut-off values and compared the effect of RFA and SR on the survival outcomes of HCC with a single lesion.

## Materials and Methods

### Data Source

Data was retrieved from the National Cancer Institute's Surveillance, Epidemiology, and End Results (SEER) database between 2004 to 2015 using SEER^*^Stat 8.3.5. The SEER database provides information on cancer statistics in an effort to reduce the cancer burden among the U.S. population. The information on type of cancer, tumor size, alpha-fetoprotein (AFP), marital status, gender, age, race, differential degree, survival time, survival status, treatment type of primary cancer, and vascular invasion were retrospectively collected.

### Inclusion and Exclusion Criteria

Patients were enrolled into this study if they met the following inclusion criteria: (1) a histological diagnosis of HCC with ICD-O-3 code 8170; (2) 18 years of age or older; (3) follow-up time longer than 3 months; (4) only one lesion measuring <5 cm in size; (5) absence of intrahepatic vascular invasion; (6) underwent RFA or SR. The exclusion criteria included: (1) not the first tumor (occurring simultaneously with or following another tumor); (2) no known survival related information; (3) presence of intra- or extra-hepatic metastases.

### Propensity Score Matching (PSM)

Because this is a retrospective study, the included patients were not randomly distributed between RFA and SR group. The unbalanced patient characteristics may result in selection bias, which can distort the real impact of RFA or SR on patients' outcome. To reduce this effect, we first calculated the propensity score using logistic regression modeling of the probability of a patient undergoing RFA or SR on the basis of age, gender, race, marital status, differentiate degree, tumor size, and AFP. Then we used the propensity score to match patients who underwent RFA or SR at a 1:1, 1:2, and 1:3 fixed ratio with no replacement, respectively. In the whole analysis, we used the method of the nearest available matching with the caliper of 0.05. After matching, standardized difference was generated and the value <0.1 was taken as an indication of the covariates which were well balanced between the two groups.

### Multiple Imputations

To alleviate potential biases caused by the missing values in covariates, multiple imputations (MI) method was used with the **mice** function from the **mice** R package. This procedure starts with building a regression model for target variables with missing values based on all other variables. Through this approach, we created 5 sets of complete datasets and then analyzed them using different statistical methods.

### Statistical Analysis

In this study, OS and cancer-specific survival (CSS) were defined as the main outcome. Categorial variables were expressed as frequency (percentages) and evaluated using the χ^2^ test. The Kaplan-Meier method was used to generate OS and CSS. The survival difference was tested by a log-rank test. To identify potential prognostic variables, Cox univariate analysis was performed and any variables with *p*-values smaller than 0.2 were subsequently included in the Cox multivariate analysis. The results were reported as hazard ratios (HRs) with their 95% confidence intervals (CIs). In addition, death due to causes other than HCC was considered to compete with the event of interest, which may underestimate the incidence of CSS. Therefore, when we estimated the cumulative cancer-specific mortality, death due to other causes needed to be taken into account. In order to examine the association of HCC with mortality, the Fine-Gray proportional hazard models were used and the results were represented as subdistribution hazard ratios (sdHRs) and their 95% CIs. A sdHR of 1 implies no association, an sdHR <1 implies a decreased risk compared with the reference category, and a sdHR >1 implies an increased risk compared with the reference category.

To make our conclusions more robust, sensitivity analyses were performed including deletion of missing values and PSM at different ratios (detailed in the above description). All the statistic tests were two-sided. A *p*-value smaller than 0.05 was regarded as statistically significant. All the above analyses were performed using R software version 2.15.3 (R Foundation for Statistical Computing, Vienna, Austria).

## Results

### Baseline Characteristics

A total of 1,985 eligible patients were enrolled in this study, of which 934 were treated with RFA and the others were treated with SR. The median follow-up period of patients in the RFA group was 30 months (range 15–53 months) compared with 34 months (range 16–61 months) in the SR group. The gender and age were similar between the two groups. More patients were married in the RFA group. Fifty-four percent of patients in the RFA group had AFP positive compared with 46% in the SR group. The number of tumors with size smaller than 3 cm were higher in the RFA group. In addition, the SR group tended to have more patients with relatively poorly differentiated tumors. More detailed information can be found in Table 1.

**Table 1:** Baseline demographic and clinical characteristics.

**Variables**	**RFA (*n* = 934)**	**SR (*n* = 1,051)**	***p***
**Age**			
≤ 65	596 (63.81)	690 (65.65)	0.418
>65	338 (36.19)	361 (34.35)	0.418
**Gender**			
Male	701 (75.05)	763 (72.6)	0.234
Female	233 (24.95)	288 (27.4)	0.234
**Race**			
White	563 (60.28)	564 (53.66)	<0.001
Black	118 (12.63)	124 (11.8)	<0.001
Others	247 (26.45)	359 (34.16)	<0.001
Unknown	6 (0.64)	4 (0.38)	<0.001
**Marital status**			
Married	504 (53.96)	626 (59.56)	0.009
Unmarried	404 (43.25)	386 (36.73)	0.009
Unknown	26 (2.78)	39 (3.71)	0.009
**Grade**			
Well differentiated	257 (27.52)	269 (25.59)	<0.001
Moderately differentiated	256 (27.41)	502 (47.76)	<0.001
Poorly differentiated	66 (7.07)	162 (15.41)	<0.001
Undifferentiated	2 (0.21)	13 (1.24)	<0.001
Unknown	353 (37.79)	105 (9.99)	<0.001
**Tumor size (cm)**			
≤ 2	216 (23.12)	219 (20.84)	<0.001
≤ 3	379 (40.58)	332 (31.59)	<0.001
≤ 5	339(36.30)	500 (47.57)	<0.001
**AFP**			
Positive	509 (54.50)	488 (46.43)	<0.001
Negative	279 (29.87)	325 (30.92)	<0.001
Unknown	146 (15.63)	238 (22.65)	<0.001
Follow-up time (month)	30 (15.53)	34 (16.61)	0.001

*RFA, radiofrequency ablation; SR, surgical removal*.

### Comparison of Survival Outcomes Before Matching

**Figure 1:**
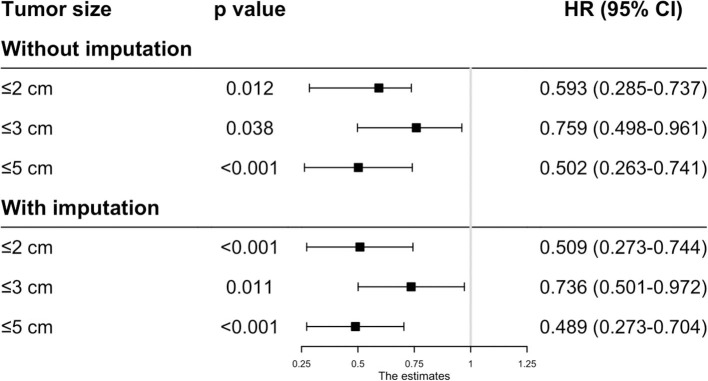
The effect of RFA and surgery on HCC patients' OS with different tumor size before PSM.

**Figure 2:**
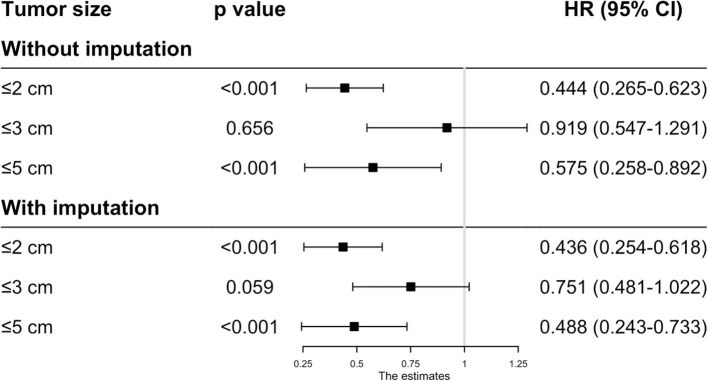
The effect of RFA and surgery on HCC patients' CSS with different tumor size before PSM.

Before matching, the patients in the RFA group had poorer OS and CSS than those in the SR group regardless of the tumor size. On multivariate analysis, a worse OS (HR: 0.593, 95% CI: 0.285–0.737, *p* = 0.012) and CSS (HR: 0.444, 95% CI: 0.265–0.623, *p* < 0.001) was observed in patients with RFA with tumors ≤ 2 cm before MI (Figures 1, 2). For tumors measuring 2.1–3 cm, the CSS tended to be similar in patients undergoing RFA compared with those receiving SR (HR: 0.919, 95% CI: 0.547–1.291, *p* = 0.656), while the OS is still better in SR group than in RFA group (HR: 0.759, 95% CI: 0.498–0.961, *p* = 0.038). When the tumor size exceeded 3 cm, the SR group had a higher OS (HR: 0.502, 95% CI: 0.263–0.741, *p* < 0.001) and CSS (HR: 0.575, 95% CI: 0.258–0.892, *p* < 0.001) than the RFA group. Furthermore, the competing risk model was built with death caused by cancer-unrelated diseases as a competing event (**Figure 5**). For tumors measuring 2.1 to 3 cm, patients receiving RFA treatment had a similar risk of cancer-related mortality compared to those undergoing SR, with SHR of 0.842 (95% CI: 0.627-1.130). However, the outcome of the SR group was more favorable then the RFA group with tumors measuring either 3.1 to 5 cm (HR: 0.615, 95% CI: 0.451–0.839, *p* = 0.002) or ≤ 2 cm (HR: 0.484, 95% CI: 0.333–0.703, *p* < 0.001).

Because the results may be affected by the variables with missing values, MI was applied to impute the missing values and the complete data was then generated. In this analysis, we found that the patients undergoing RFA had a better OS with tumors ≤ 2 cm (HR: 0.509, 95% CI: 0.273–0.744, *p* < 0.001), ≤ 3 cm (HR: 0.736, 95% CI: 0.501–0.972, *p* = 0.011) and ≤ 5 cm (HR: 0.489, 95% CI: 0.273–0.704, *p* < 0.001) (Figure 1). However, the CSS time for RFA was similar to SR for tumors measuring 2.1–3 cm (HR: 0.751, 95% CI: 0.481–1.022, *p* = 0.059). For those whose tumors measured 3.1–5 cm (HR: 0.488, 95% CI: 0.243–0.733, *p* < 0.001) or ≤ 2 cm (HR: 0.436, 95% CI: 0.254–0.618, *p* < 0.001), no significant different was observed in CSS between RFA and SR after MI (Figure 2).

### Comparison of Survival Outcomes After Matching

**Figure 3:**
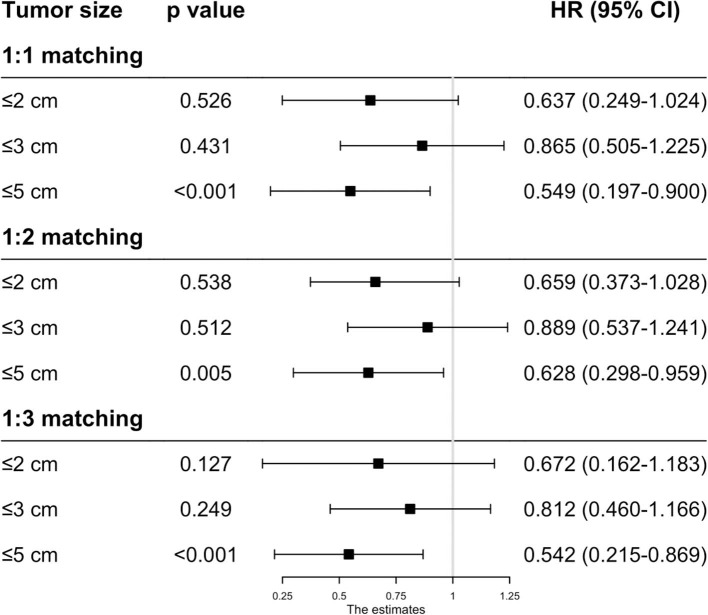
The effect of RFA and surgery on HCC patients' OS with different tumor size after PSM.

**Figure 4:**
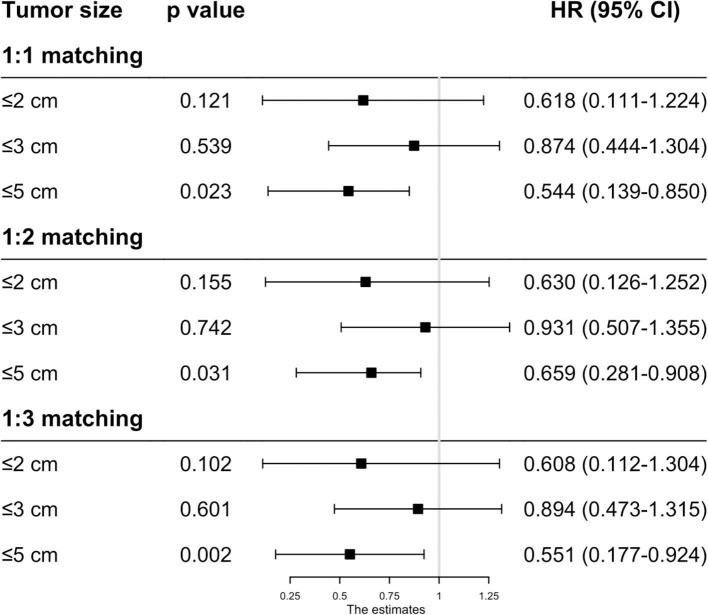
The effect of RFA and surgery on HCC patients' CSS with different tumor size after PSM.

**Figure 5:**
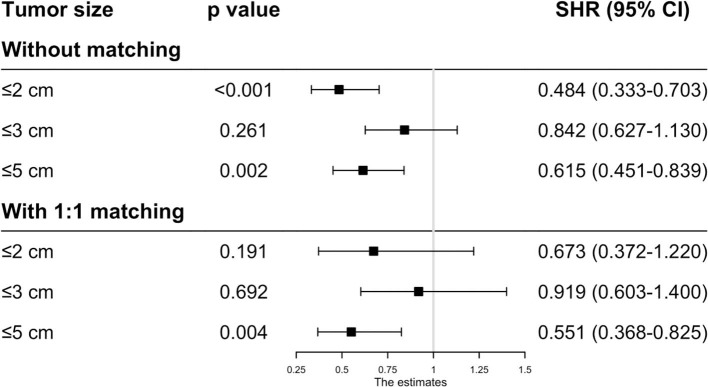
Adjusted HRs for the mortality of HCC patients receiving RFA and surgery before/after PSM.

As the baseline characteristics between the RFA and SR group were not the same in the original data, which may lead to inaccurate conclusions, we therefore performed PSM analysis to balance the covariate variables except for therapeutic options. To enhance the validity of our results, we conducted PSM at a 1:1, 1:2, and 1:3 ratio, respectively, and standard difference <0.1 was taken as an indication of well-balanced variables between the two groups. Univariate and multivariate Cox analyses were carried out stratified by tumor size. The results show that RFA and SR were correlated with similar OS (HR: 0.637, 95% CI: 0.249–1.024, *p* = 0.526; HR: 0.865, 95% CI: 0.505–1.225, *p* = 0.431) and CSS (HR: 0.618, 95% CI: 0.111–1.224, *p* = 0.121; HR: 0.874, 95% CI: 0.444–1.304, *p* = 0.539) with tumor size ≤ 2 and ≤ 3 cm (Figures 3, 4). Whereas for tumors measuring 3.1 to 5 cm, patients after SR had a significant improvement in OS (HR: 0.549, 95% CI: 0.197–0.900, *p* < 0.001) and CSS (HR: 0.544, 95% CI: 0.139–0.850, *p* = 0.023) compared with those after RFA. This result was maintained after PSM analysis at a 1:2 and 1:3 ratio. A similar trend was also observed in the Fine-Gray proportional hazard model (Figure 5).

## Discussion

The purpose of this study was to investigate the therapeutic effect of SR and RFA on HCC patients with a solitary lesion measuring ≤ 5 cm. By applying several effective sensitivity analyses, we have demonstrated that SR had a positive impact on OS and CSS of patients with tumors measuring 3.1–5 cm. However, when the tumor size ≤ 3 cm, patients had a similar survival benefit from SR as from RFA.

Our findings are in agreement with the study by Kutlu et al. (8), which is also conducted based on the SEER database. But our study differs from theirs in three aspects: (1) There are a significant proportion of patients with data missing in this database. The authors chose to delete these missing data and it may result in inaccuracy of the analyses that follow. Therefore, in order to solve this problem, we performed MI analysis in this study, which is an effective approach in dealing with missing data, and the results remained consistent before and after MI analysis. (2) Given potential confounders differed between the SR and RFA group, we also performed propensity score matching to mitigate biases caused by these unadjusted valuables. (3) In the study by Kutlu et al. (8), OS and CSS were considered as the primary event of interest. However, they did not account for the fact that this could result in a bias when using the Kaplan-Meier method in the presence of competing risks, because in this case, the competing risk events are treated as censored data. Non-cancer deaths as a competing event may mislead one to accurately estimate the real mortality rate of HCC. Thus, the Fine-Gray model was also constructed to determine whether or not the therapeutic approach was an independent prognostic factor. Through these methodological improvements, we believe the conclusions will be more reliable.

There have been some reports comparing the efficacy of RFA with SR in small, solitary HCC, however the results proved to be contradictory (2, 4, 6, 7, 13). In the analysis of patients with HCC measuring ≥3 cm, SR was shown to be superior to RFA with respect to OS and CSS in our study regardless of PSM, whereas several studies reported that the effect of RFA on HCC ≥3 cm was comparable to that of SR. For example, the results of a study from France including 281 patients with HCC measuring ≤ 5 cm have shown no survival difference between the RFA and SR group (5). In addition, another study involving 152 cirrhotic patients undergoing either RFA or SR demonstrated that these two therapies had similar survival rates for single HCC nodules measuring ≤ 5 cm (14). The discrepancy of these results may be partly due to the type of RFA device used. For example, multipolar devices, which offer better outcomes for HCC patients, have stronger capacity for destroying large tumors than multi-tined expandable monopolar devices. Therefore, some authors pointed that it could result in a bias if several kinds of devices were applied (15). In addition to different types of devices, RFA can be carried out by percutaneous, laparoscopic, or open approaches. It is reported that laparoscopic RFA (LRFA) exerted better therapeutic efficacy than percutaneous approach, especially for those lesions close to the gallbladder, stomach, colon, or other visceral structures (16, 17). So bias might also occur with the application of different RFA approaches. As the RFA probe type is unknown in this database, and multipolar devices, as a newly-invented technique, have only recently entered clinical practice, we think in our study the patients receiving monopolar RFA treatment are more numerous than those receiving multipolar treatment. We believe that is why we found patients in the SR group had a better prognosis.

With regard to HCC ≤ 3 cm, our results show that there is no significant difference in survival rate between the RFA and SR group, which is similar to several previous studies (7, 18–20). It has been proven that the advantage of SR lies in the complete removal of tumor tissue and hepatic parenchyma around the tumor, which might contain undetectable micrometastases and microvascular invasion. When the tumor is small, it is relatively less likely to have satellite nodules, and therefore, it is possible for RFA to erase the lesion. If the size of tumor exceeds 3 cm, it becomes difficult for clinicians to remove the microlesions completely using the RFA method. So, the effectiveness of RFA vs. SR for HCC (<5 cm) is expected to be different when the 3 cm cutoff value is considered.

However, according to the published reports, the efficacy of the two therapies in HCC with size 2–3 cm is quite controversial. In a work conducted by Cucchetti et al. (21), it was shown that surgery might provide a better prognosis than RFA in 2–3 cm HCC. Normally during the RFA procedure, in order to overlap target regions in a large tumor, the needle electrodes need to be placed more than one time, and thus it is not easy to reach the desired temperature throughout all the areas of the nodule (9). Therefore, the efficacy of RFA is considered to be highly size dependent. Some studies have reported that a higher incidence of local recurrence was observed in patients following RFA (22, 23). This may be explained by the fact that the procedure of thermal ablation can increase intratumoral pressure and thus promote the spread of tumor cells into the adjacent portal vein (17). Other factors such as the heat-sink effect or microscopic satellites and emboli in adjacent vasculature may also contribute to this phenomenon (19). In spite of the tendency to relapse, Hung et al. (22) found those in the RFA group still have satisfactory survival outcomes comparable to the SR group. One reason for this is that most of the patients underwent close surveillance after RFA, so the recurrent tumor is detected easily and treated completely by subsequent local ablation (22). Therefore, it is believed that the higher risk of recurrence is not a major obstacle to apply RFA as first-line treatment for solitary small HCCs. In addition, over the last decade, due to the advances in RFA devices and needle electrode technology, clinicians have been able to apply RFA to larger tumors. The current RFA system is able to destroy areas of liver parenchyma with diameters of more than 5 cm in a single application (9). Therefore, from our point of view, RFA is recommend as an effective and safe treatment option for single HCC ≤ 3 cm (24).

But one thing should arise our attention that treatment strategy is also dependent on patients' fitness condition.

Because sometimes patients' physical condition is not allowed to endure the surgical intervention. Under those circumstances, SR may not be the optimal option even if the tumor grows beyond 3 cm.

Our study has several limitations. First, due to its retrospective nature, potential bias still possibly exists. The selection bias, for example, might not be completely avoided even after careful PSM analysis. Second, our study also has limitations specific to the SEER database. Information such as underlying liver disease, liver function, the presence of portal hypertension, surgical margin status, and RFA approaches are not provided, and these variables may be different between both groups and have effects on the patients' prognosis. Additionally, indices such as international normalized ratio (INR), creatinine, and bilirubin could be filled in the database, but often such information was not submitted. As a result, the Child-Pugh or MELD Score could not be calculated for further investigation. Thus, randomized-controlled studies in multiple centers are necessary to help further clarify this question.

## Conclusion

In summary, by using PSM analysis to mitigate the selection bias between the RFA and SR group, patient outcomes were reanalyzed using comparable clinicopathologic characteristics. As a result, we have better defined the actual effectiveness of RFA and SR in treating solitary HCC. We have verified our results with further analysis by the use of multiple imputations of missing data and a competing risk model. We found that OS and CSS were similar between both treatments with tumors ≤ 3 cm, and thus both RFA and SR are highly recommended in this situation. While surgery may be a superior treatment option with better long-term outcome than RFA in patients with tumors measuring 3.1–5 cm.

## Data Availability Statement

All datasets generated for this study are included in the article/supplementary material.

## Ethics Statement

The study was approved by the Ethics Committee of Shanghai Ninth People's Hospital. As this is a retrospective study in nature, the informed consent was not required in this study.

## Author Contributions

LZ, C-LS, C-HZ, and J-YL contributed to data acquisition. LZ, C-LS, and J-YL performed the statistical analysis and prepared the manuscript. LZ and C-HZ drafted this manuscript. X-LQ and ML supervised the study. All authors read and approved the final manuscript.

### Conflict of Interest

The authors declare that the research was conducted in the absence of any commercial or financial relationships that could be construed as a potential conflict of interest.

## Abbreviations

HCC: Hepatocellular carcinoma
RFA: Radiofrequency ablation
SR: Surgical resection
OS: Overall survival
DFS: Disease-free survival
SEER: Surveillance Epidemiology and End Results
AFP: Alpha-fetoprotein
PSM: Propensity score matching
MI: Multiple imputations
CSS: Cancer-specific survival
Cis: Confidence intervals
HRs: Hazard ratios
sdHRs: Subdistribution hazard ratios
INR: International normalized ratio.
